# Annotations

**Published:** 1907-05-18

**Authors:** 


### Annotations.

### Wasp Waists Again.

With the tight bodice comes the wasp waist. That has been fashion's rule from the beginning, and doubtless will be to the end. But during the last fifteen years influences have arisen, which, quite apart from fashion, will make it difficult for girls to achieve that dubious adjunct to beauty. Bicycling, gymnastics, hockey, have developed our girls so that they could not, even if they would, crush their bodies in the way that their grandmothers did. But, in spite of alarmists, we do not think that wasp waists are at present so numerous as to endanger the health of the bulk of our women. Tight-lacing, to be successful, must be begun when a girl is young, and no one who is familiar with the temper of our school-girls to-day will readily believe that they will prove as wax in the hands of dressmakers, corsetières, or others who want to constrict them in any uncomfortable degree. Good, well-shaped corsets can be bought as cheaply now as bad and uncomfortable ones were a generation ago, and if the lines of gown and corset are good, a waist that bears a decent proportion to its owner's height and breadth may still look slender. There are many slim and graceful women, who, if put to the test, would prove to have quite a reasonable waist. Moreover, it is whispered that in the "sizes" of gloves and shoes the same number does not imply the same measurements as it did in bygone years, and ladies who used to wear 6½ gloves find that to-day a 6 is large enough for a hand that shows no sign of having grown smaller. Something of the same kind may be going on in the corset world, and a conventional 17 may not mean just the 17 inches which its wearer fondly thinks. On the whole, we are not alarmed about the threatened re-introduction of the wasp waist. That there are, as there always have been, simpletons who sacrifice health and comfort to false ideas of beauty, we doubt not, but they are a very small percentage of our womenkind.

### Cardiac Disease in Early Life.

At a recent meeting of the Society for the Study of Disease in Children a number of examples of cardiac disease in young patients that were exhibited led to a discussion in which some interesting points were raised. In one patient, a boy of some 12 years, there was complete dextrocardia, and the facts of the case, and a statement by the mother of some medical observations made on the child in infancy, justified the view that this was a congenital condition, though the evidence showed that the liver and other viscera were not transposed. The special feature of the case, however, was the existence of presystolic and systolic murmurs heard most distinctly over the cardiac impulse situated just outside the right nipple line, and conducted round the right side of the chest. From these facts it was argued that in all probability these murmurs were produced at the mitral valve, which in a case of congenital dextrocardia—that is, of true transposition and not mere displacement of the heart—must be situated at the right auriculo-ventricular orifice. The presence of murmurs at the right apex and conducted to the right were held to support the view that the case was one of true transposition. Unfortunately, repeated attempts to obtain a skiagram with a view to determine whether the arch of the aorta was on the right or left side had proved unsuccessful. In a second case a little girl with mitral disease awaked one morning to find her left upper limb paralysed, and it was suggested that this was due to embolism affecting the cerebral cortex; some of the speakers rejected this view in favour of the diagnosis of thrombosis. A third patient, in addition to evidences of congenital disease of the aortic valve, had a continuous humming noise immediately to the right of the sternum; the interpretation of this seemed very uncertain, but one speaker suggested that it might be due to some abnormal condition of the veins in the upper thorax.

### Medical Titles.

Every now and then a discussion, sometimes not unattended with bitterness, breaks out in reference to the correct styles and titles which should be applied to various groups of medical practitioners. Does a certain qualification entitle a man to call himself a physician? Or is another a warrant for the use of the term "Doctor"? These disputes doubtless appeal mostly to minds of a juvenile order, but, as may be seen from recent contributions to some of our contemporaries, there are quite a number of graduates, even of the Metropolitan University, who appear to consider that their chief claim to distinction resides in the fact that they at one time in their lives managed to pass a moderately difficult examination. Whether such success was a decided one or was perilously near the margin of failure is a question not discussed, nor is any reference made to the amount of artificial or special aid which had to be invoked as a necessary preliminary to the examination contest. Now all these questions may well be regarded as trivial by men whose sense of individual values has been informed by experience and who recognise that personal worth has little to do with alphabetical decorations. Yet it is perhaps necessary to allow something for human peculiarities, and it is certain that in the disputes to which reference is here made, personal feeling in an acute form is sometimes engaged. To meet the position it has been suggested that professional recognition shall be extended to the proposal to apply the term "Doctor" to every qualified medical practitioner. This, of course, excites opposition in certain quarters, and as an alternative it is now proposed that all medical practitioners shall be content with the term "Mr." Personally, we think there is much to be said for this view. It has its parallel in the practice of the legal profession, it avoids disputes, and it satisfies the demands of courtesy. Further, it allows a medical practitioner to escape in his periods of relaxation from the atmosphere of his technical work. Why should the medical man not be allowed to take his holiday or to attend a dinner party, free from the announcement of his daily professional activities?

